# Peer support for eating disorders: a pilot open trial of peer support for children and adolescents with eating disorders

**DOI:** 10.1186/2050-2974-2-S1-O64

**Published:** 2014-11-24

**Authors:** Stephanie Wade, Hunna Watson, Jemma Caswell, Julie Purcell

**Affiliations:** 1 School of Psychology, University of Western Australia, Perth, Australia; 2 Eating Disorders Program, Specialised CAMHS, Child and Adolescent Health Service, Princess Margaret Hospital, Perth, Australia; 3 Body Esteem Program, Perth, Australia

## 

The current pilot study aimed to evaluate the effectiveness of a brief peer support intervention for adolescents with eating disorders, run within an established specialist hospital service. Peers are defined in this context to reflect individuals who have experienced overcoming an eating disorder. The peer support intervention was designed to run concurrently with the hospital program, with focus on removing the additional barriers to recovery, such as low motivation for recovery, that are not specifically targeted by traditional health systems. Participant groups of inpatients, day-patients and outpatients each attended two structured group sessions, and completed pre- and post-group questionnaires. Quantitative and qualitative analyses comparing pre- to post-group ratings revealed a significant decrease in participant's feelings of stigma associated with having an eating disorder; a significant increase in hope and motivation for recovery and a better future; and improved trust and acceptance of the hospital treatment they were currently receiving. The presentation will also cover the experiences of the peer workers who facilitated the groups, and the attitudes of the hospital staff towards peer support.

This abstract was presented in the **Peer Support** stream of the 2014 ANZAED Conference.

